# Transgender Patient Preferences When Discussing Gender in Health Care Settings

**DOI:** 10.1001/jamanetworkopen.2023.56604

**Published:** 2024-02-19

**Authors:** Vern Harner, Megan Moore, Boi Casillas, Jess Chrivoli, Amaranta Lopez Olivares, Erin Harrop

**Affiliations:** 1 School of Social Work, University of Washington, Seattle; 2 Now with School of Social Work and Criminal Justice, University of Washington, Tacoma; 3 School of Social Work, Harborview Injury Prevention and Research Center, University of Washington, Seattle; 4 Graduate School of Social Work, University of Denver, Denver, Colorado

## Abstract

**Importance:**

Transgender (trans) patients report frequent discriminatory and harmful experiences in health care settings; gender-affirming, competent health care improves trans individuals’ health and well-being.

**Objective:**

To identify factors influencing trans patients’ decision-making regarding sharing gender-related information with providers (clinicians, health care organizations, health care systems) and trans patient preferences for gender-related questions, as well as the subsequent impact on health care experiences.

**Design, Setting, and Participants:**

A qualitative, interpretivist phenomenological study was conducted via telephone or video (using Zoom) in English and Spanish from August 10 to September 11, 2020. Participants were recruited via trans-specific social media groups and email listservs. Interviews were transcribed verbatim and analyzed from October 2020 to January 2022 using thematic analysis by a majority trans research team.

**Main Outcomes and Measures:**

Semistructured interviews explored patient health care experiences and preferences.

**Results:**

Participants included 27 trans and/or nonbinary adults (mean [SD] age, 30.9 [10.4] years; range, 21-69 years) from 13 US states. Four themes were identified: (1) impact of provider behaviors, (2) engaging in relational risk assessment, (3) receiving affirming vs medically competent care, and (4) “how are you going to fit [me] into your system?” The first theme reflected the impact of provider actions, including level of flexibility in care and interpersonal interactions, ability to explain medical relevance of gender-related questions, and preparedness to work with trans patients. Second, respondents weighed a variety of implicit or explicit safety cues when deciding whether and how to share information with providers. Third, participants spoke at length about affirming and medically competent care, noting that they have had to prioritize one over the other. Last, participants reported their needs often being at odds with health care system norms and standards of care.

**Conclusion and Relevance:**

In this qualitative study of trans patient preferences when discussing gender-related information with providers, it was found that equitable, gender-affirming, and competent health care requires flexibility and responsiveness to patient preferences. It requires provider humility and engagement, as well as institutional policies that support competent and gender-affirming spaces. Future research could identify additional roles that providers, administrators, and systems can play in ensuring competent care for trans patients.

## Key Points

**Question:**

How do transgender patients prefer to discuss gender-related information in health care settings, and what is the subsequent impact on health care experiences?

**Findings:**

In this qualitative study that included 27 transgender and/or nonbinary adults, 4 themes were identified. These themes were (1) impact of provider (clinicians, health care organizations, health care systems) behaviors, (2) engaging in relational risk assessment, (3) receiving affirming vs medically competent care, and (4) “how are you going to fit [me] into your system?”

**Meaning:**

This study suggests that multilevel interventions are required for gender-affirming, competent health care for transgender patients; specific provider- and hospital-level recommendations are provided.

## Teaser

This qualitative study identifies factors influencing trans patients’ decision-making regarding sharing gender-related information with providers and trans patient preferences for gender-related questions, as well as the subsequent impact on health care experiences.

## Introduction

Although gender-affirming health care improves the well-being of transgender (trans) individuals (ie, transgender, nonbinary, those with culturally specific identities, and others), trans patients continue to report frequent discriminatory and unskilled treatment in health care spaces.^1,2,3,4,5,6,7,8,9^ These experiences are associated with suicidality, substance use, and other negative outcomes.^10,11,12,13^ Consequently, trans patients sometimes withhold gender-related information to avoid discrimination.^14^ Furthermore, discriminatory policies creating barriers to health care access for trans people are in flux across the US.^15^ Multiply marginalized (eg, Black individuals, Indigenous individuals, members of other racial or ethnic minority groups, disabled individuals, people with lower socioeconomic status) trans people are already more likely to use emergency departments for primary care, which is associated with worse health outcomes compared with visiting primary care physicians.^16,17^ Although these experiences are increasingly well documented, there is a dearth of scholarship investigating specific aspects of gender-affirming health care practices. Given these challenges, we posed the following research questions: (1) What influences patients’ decision-making regarding sharing gender-related information with health care providers (clinicians, health care organizations, health care systems)? (2) How do patients want to be asked gender-related questions? (3) How does provider understanding of gender impact patients’ health care experiences?

## Methods

### Setting, Participants, and Study Design

From August 10 to September 11, 2020, we recruited (eg, via social media, listservs) and interviewed 27 trans adults. Additional eligibility criteria included having accessed health care in the past 5 years and ability to complete a telephone or video interview in English or Spanish. Recruitment materials advertised that interviews would be conducted by trans interviewers and that the majority of the research team was trans. All study procedures were approved by the University of Washington institutional review board, and this qualitative study followed the Consolidated Criteria for Reporting Qualitative Research (COREQ) reporting guideline. Participants provided written informed consent.

### Procedures and Data Collection

After signing informed consent, participants completed a demographic questionnaire and a 45- to 90-minute semistructured interview^18^ conducted on video (Zoom) by 1 of 2 trans research team members (V.H. and A.L.O.). Interviews explored participants’ experiences receiving health care (general, trans-specific, and emergency) and how providers in various care settings can be more inclusive and affirming. At the conclusion of their interview, participants were given the opportunity to reflect on and/or redact any part of their responses. Participants received a $75 gift card for participation.

### Data Analysis

Data analysis took place from October 2020 through January 2022. We used an interpretivist phenomenological approach, which focuses on developing a rich understanding of participants’ lived experiences, rather than seeking generalizable findings.^19,20,21^ Aligned with the interpretivist phenomenological approach, the team completed regular memorandas and analytic discussions.^22^ Interviews were audio recorded, transcribed verbatim, validated, deidentified, and stored in REDCap.^23,24^ Transcripts were analyzed using Dedoose, version 9.0.17 (SocioCultural Research Consultants, LLC),^25^ following thematic analysis procedures.^26^ Methods for extracting and highlighting themes from the data included several strategies to increase trustworthiness of the results. One of us (V.H.) created an initial code list via inductive coding of 3 transcripts and a list of a priori codes.^19,26^ Next, the team discussed and refined codes based on consensus. Transcripts were then coded by 3 team members (V.H., B.C., and J.C.) across the following domains: health care spaces or types, experiences of services, structural or individual oppression, context, and suggestions. Biweekly meetings were held to discuss coding, describe and interpret themes, and identify salient quotes.

## Results

Participants included 27 trans adults (mean [SD] age, 30.9 [10.4] years; range, 21-69 years) from 13 US states (Table 1). Most participants were White (n = 22) and nonbinary (n = 18). Additional demographic characteristics are found in Table 2. Four major themes (Box) were identified: impact of provider behaviors, engaging in relational risk assessment, receiving affirming vs medically competent care, and “how are you going to fit [me] into your system?”

**Table 1.** Participant Overview

Patient No./age range (y)/gender identity	Gender expression	Sexual orientation	Disabled or chronically ill	Race and ethnicity	US Region	Income, $	Relationship status
1/25-29/Trans*butch	Butch, masculine	Queer	No	White	Northeast	25 000-29 999	Partnered or married to 1 person
2/25-29/Transmale	Male	Queer	No	White	Midwest	40 000-49 999	Dating 1 person
3/25-29/Transgender and genderqueer	Masc of center and chaotic	Queer	Yes	White	Midwest	Prefer not to say	Single
4/20-24/Gender nonconforming	Genderfuck femme	Gay	Yes	Asian, Middle Eastern, and White	Southeast	1-5000	Partnered or married to 1 person
5/25-29/Genderqueer	Surfer or ski bro (depending on season) but add makeup and earrings	Queer	No	White	Midwest	10 000-12 499	Dating 1 person
6/25-29/Nonbinary trans	Decline to respond	Queer	Yes	White	Southeast	20 000-24 999	Single
7/30-34/Nonbinary trans woman	Not butch or femme or androgynous	Queer	Yes	White	Northwest	15 000-17 499	Dating multiple people
8/30-34/Agender, nonbinary, sometimes trans	Androgynous some days, “whatever” other days	Bisexual or pansexual	Yes	White	Northwest	25 000-29 999	Partnered or married to 1 person
9/20-24/Transmasculine	Crunchy gay	Queer	No	White	Northeast	10 000-12 499	Dating and partnered to multiple people
10/20-24/Nonbinary	Varying	Queer	Yes	White	Midwest	10 000-12 499	Partnered or married to 1 person
11/30-34/Transmasc and nonbinary	Hard femme to androgynous	Queer	Decline to respond	White	Northeast	20 000-24 999	Partnered to 1 person and dating multiple people
12/20-24/Nonbinary	Sometimes femme, sometimes androgynous	Queer, demisexual, gray-a	Yes	White	Midwest	25 000-29 999	Single
13/20-24/Male	Male	Pansexual	No	White	Midwest	5000-7499	Dating 1 person
14/30-34/Nonbinary trans man	Genderqueer masc of center	Bisexual, queer	Yes	Latinx	Southwest	No income	Partnered or married to 1 person
15/40-44/Trans male	Masculine	Pansexual	Yes	White	Southwest	50 000-59 999	Partnered or married to 1 person
16/25-29/Transmasculine nonbinary	Masculine; “I am generally read as a cis man, albeit a queer one”	Aromantic asexual	Yes	White	South	12 500-14 999	Single
17/65-69/“I look, sound, and pass as male”	Gay or bisexual male	Gay or bisexual	No	White	Northwest	30 000-34 999	Single
18/20-24/Transmasculine nonbinary	Gender neutral or masculine	Bisexual; “I’m on the asexual and aromantic spectrums but prefer not to use the specific microlabels that could describe my orientation”	Yes	White	Northeast	5000-7499	Dating 1 person
19/40-44/Nonbinary	Masculine of center	Pansexual and romantic	Yes	Latinx	Northwest	25 000-29 999	Partnered to 1 person and dating multiple people
20/35-39/Nonbinary trans male	“My expression or aesthetic are frequently read as male or masculine”	Queer or gay	Yes	White	Northwest	5000-7499	Single
21/30-34/Nonbinary	Preferred femme but androgynous for safety	Queer	Yes	White	Southeast	15 000-17 499	Single
22/40-44/Female	Femme	Lesbian	Yes	White	Decline to respond	15 000-17 499	Partnered or married to 1 person
23/20-24/Nonbinary	Androgynous, kind of masc, kind of fem	Queer	Decline to respond	Asian	Southeast	15 000-17 499	Single
24/35-39/FTM	Masculine leaning	Queer with more attraction currently to men	No	Asian and White	Northwest	≥150 000	Single
25/44-49/Male	Masculine	Queer	No	Latinx	Northwest	100 000-149 999	Partnered or married to 1 person
26/25-29/Nonbinary, agender	Femmeish	Bisexual or queer	Yes	Latinx, Middle Eastern, and White	Northwest	1-5000	Partnered or married to 1 person
27/20-24/Nonbinary	Decline to respond	Gay	Decline to respond	Middle Eastern	Northwest	30 000-34 999	Single

Abbreviation: FTM, female to male.

**Table 2.** Participant Characteristics

Characteristic	No. (%) (N = 27)
Collapsed gender identity	
Nonbinary	18 (66.7)
Man or trans masc	7 (25.9)
Woman or trans fem(me)	2 (7.4)
Black, Indigenous, or other racial or ethnic minority group	5 (18.5)
Disabled and/or chronically ill	16 (59.3)
Annual individual income, $ (range, 0 through >150 000)	
<40 000	24 (88.9)
Has health insurance	25 (92.6)
Has PCP	17 (63.0)
Visits PCP once per year	3 (11.1)
Visits PCP 2-5 times per year	11 (40.7)
Visits PCP >5 times per year	3 (11.1)
Visited urgent care in past 5 y	17 (63.0)
Visited ED in past 5 y	12 (44.4)
Hospitalized in the past 5 y	9 (33.3)

Abbreviations: ED, emergency department; PCP, primary care physician.

Box. **Overview of Themes**

**Impact of Provider Behaviors**

Individual providers’ actions had significant positive and negative impact on participants’ experiences, depending on how attuned, informed, and prepared the provider was.

**Examples**

I tried this [provider] because it [the health setting] was supposed to be like a queer-friendly place and she said, “Yeah, no problem. I have a place for pronouns and you know, trans men come in here all the time to take care of their lady parts.” And I said, “All right, we’re okay. We’re good. We’re done.” To me it flagged immediately… you don’t understand. It’s still like a gender essentialist view, right? Like a biological and sex essentialist view… and so regardless of how nice you are to use our language, you don’t get it. (Participant 23)
I went on birth control for a little bit and the whole language around [it] made me super uncomfortable, but it was a thing that I needed to have. So, I feel like procedures should be very nongendered. Like, “Oh, you’re on this pill for this reason. Here’s some side effects.” …Not, “This is a woman pill for women things.” (Participant 13)
… Ask when it’s relevant, and if …something could be relevant if the person’s trans, that means you have to ask everyone because you can’t tell who’s trans. (Participant 20)
Your transness is always relevant if you’re in danger or if the care you’re about to receive is going to be impacted. And that’s [something of] relevance you have really no control over. (Participant 4)
Any efforts to include more collection of, like, gender and sexual minority data needs to be accompanied with the necessary training and protocols and process because, if you’re not doing that and having that set up before you even ask, you’re asking marginalized people to disclose things that could get them in trouble or put in harm’s way. (Participant 23)

**Engaging in Relational Risk Assessment**

This theme evolved from respondents sharing how they weighed a variety of implicit and explicit safety cues when deciding what and how to share information with their providers in a variety of health care settings. Participants noted how vulnerable health care spaces felt for them, with several directly mentioning the power imbalance between patient and provider.

**Examples**

Until I know that there are strict regulations in place to protect me, which there are not, um, I would rather be misgendered than have my life be at risk. (Participant 4)
The benefit to having a conversation with a provider is to gauge their reaction… and get a good sense of if they’re going to be a good fit for me or not. …I mean, seeing [inclusive gender options] on the form is like a flag …it’s flagging, right? If, when they come in and introduce themselves, they don’t default to my birth name—which has happened even with providers that tout themselves as trans-friendly—or not using a prefix that I don’t use, especially when it’s in a waiting room and it’s public …those kinds of things immediately signal that you read the form, you’ve got it and you’re trying to get off on the right foot with me. (Participant 11)
With my primary care guy, I haven’t had this conversation. I’m 90% sure it’d go fine, but at the same time, I really love the care I get there. And I think that’s what makes me hesitate to even mention it at this point. (Participant 19)
I want to have important conversations about, like, hormones or about, um, getting top surgery, but I don’t want to jeopardize getting good diabetic treatment. (Participant 10)

**Receiving Affirming vs Medically Competent Care**

Gender- or trans-affirming, transition-related, and medically competent care are all distinct and subjective, although at times overlapping aspects of health encounters. Participants spoke at the most length and detail about affirming and medically competent care, noting that they have had to, at times, choose to prioritize one type of care over another.

**Examples**

I’m disabled, and I often find myself in situations where I need to decide… is it important that I point out that I’m being misgendered and I feel really upset by that? Or is it important that I receive the medical care that I need? And I feel like I’m always balancing that in general… when I’m going into anaphylactic shock, it just doesn’t matter. The fact that I’m trans doesn’t matter, because epinephrine works no matter what, and that’s what I need. You need to know that I’m a hard stick and that my right arm is probably the best place to try. That’s the information we need at the moment. (Participant 10)
… Life has just turned completely upside down and I was in a lot of pain. It was almost impossible to self-advocate. And if they had used my pronouns when referring to and talking with me that would have just brought a little bit more ease to that very difficult situation… it feels important to have it recognized, especially in the urgent care and [emergency department] settings to help with not making it more traumatic than it already is. (Participant 19)
I think the bottom line for medical providers is if they’re well informed and they really see us for who we are, we’ll know …and if they don’t …we’ll know. I think that trans people are really good at sniffing out who’s an ally and who’s not, and who’s trying to do good by us and maybe who’s learning but still figuring it out. (Participant 11)
… The doctor I am currently going to was the first person who explained to me what you’re really checking for when you do the blood work at the different phases after a shot… and that instantly gave me a good feeling because he told me something I didn’t know… because it’s kind of tiring when you deal with providers where you’re clearly the expert. (Participant 16)

**“How Are You Going to Fit [Me] Into Your System?”**

Participants spoke to their needs as patients often being at odds with the health care system status quo. Whether that meant not fitting neatly into a prescribed identity label (ie, gender assigned at birth) or the health care system prioritizing very different tasks or interventions, respondents reported that their needs simply did not fit into current medical practices, policies, and procedures.

**Examples**

These 2 boxes (man or woman) have just come to represent so much more information than they really do. (Participant 20)
Not all nationalities have the same idea of gender. The country that I’m from, there’s the oldest recorded—what the Western culture calls—trans community in history, …they’re not trans, these are like blessed beings, you know? It’s who you are. There’s a culture behind it. It’s hard to explain, but how are you going to fit that into your system? (Participant 4)
I don’t know why fucking cis people love the word *preferred* …like they “prefer” oat milk. …I just think the language is trash and it’s rooted in transphobia. Just ask what their pronouns are. (Participant 11)
I think we get really, really caught up in these, like, “Are we asking the right questions? Are we getting it right? Can you please tell us that we’re doing the right thing?” more so than some of the deeper, real, real shit that people need to. So again, that’s not necessarily helpful. We need, like, a larger trans health, um, reckoning… because this is the easy stuff. (Participant 9)

### Impact of Provider Behaviors

Provider interactions significantly impacted with participants; the nature of the impact depended on how attuned to and informed the provider was. Participants highlighted the importance of providers recognizing risks inherent in patients outing themselves: “[Collecting these] data needs to be accompanied with the necessary training and protocols and process because… you’re asking marginalized people to disclose things that… put [us] in harm’s way” (participant 23). Others mentioned the risk of receiving subpar care or being refused care: “Your transness is always relevant if you’re in danger or the care you’re about to receive is going to be impacted” (participant 4).

Participants suggested that gender-related information be asked when medically relevant, which requires careful screening: “Ask when it’s relevant, and if …something could be relevant if the person’s trans, that means you have to ask everyone because you can’t tell who’s trans” (participant 20). Participants wanted clear, specific justifications for collecting information (eg, legal name for insurance purposes), biometric data (eg, hormone levels, anatomy), and explanations for how information would be shared and stored: “I thought they were asking me a completely unnecessary personal question, [but] it was shockingly relevant [to ask about gender]” (participant 20).

Participants expressed concerns about the potential for receiving harmful care, particularly in emergency department settings where trans patients are unable to avoid transphobic providers. Participants feared losing access to hormone replacement therapy, experiencing microaggressions, receiving subpar care, or even being refused emergency care. Unnecessarily gendered language in any health care setting (ie, “women’s health”) prompted dysphoric feelings and fears of care refusal. For example, participant 13 shared how the language regarding birth control made them “super uncomfortable”: “Procedures should be very nongendered. Like, ‘Oh, you’re on this pill for this reason. Here’s some side effects.’ …not, ‘This is a woman pill for women-things.’” Similarly, participant 23 explained how unaffirming it felt when a provider told them that “trans men come in here all the time to take care of their lady parts.’” This statement created an immediate atmosphere of distrust with a provider who had purported to be “queer friendly”: “Regardless of how nice you are to use our language, you don’t get it.”

### Engaging in Relational Risk Assessment

Participants reported feeling vulnerable in health care spaces and needing to engage in subsequent relational risk assessment, due to power imbalances between patient and provider and potentially being refused care. For example, participant 16 viewed physicians as the “expert,” reporting, “people will feel psychologically compelled to answer [invasive questions] because of the power dynamic.”

When assessing risk, participants weighed implicit and explicit safety cues. Explicit cues included direct experiences (eg, providers introduced themselves with pronouns, chosen names were solicited and seamlessly used, prior experiences were shared). Explicit cues occurred in both verbal and written formats. Participants referred to these cues as “flagging” or “signaling.” Participant 11 described “gauging [provider] reactions” in conversations and flagging language in paperwork to intuit provider fit: “If… they don’t default to my birth name—which has happened even with providers that tout themselves as trans friendly—or not using a prefix that I don’t use… [that] immediately signal[s] that you’ve got it and you’re trying to get off on the right foot with me.”

Implicit cues included indirect experiences (eg, organizational policies, LGBTQ [lesbian, gay, bisexual, trans, queer] materials on websites or in waiting rooms). To determine disclosure risk, participants often relied on these implicit cues: “Until I know that there are strict regulations in place to protect me… I would rather be misgendered than have my life be at risk” (participant 4). Although not all religious-based institutions are inherently unsafe for trans individuals, in the absence of trans-affirming cues, participants doubted if competent care would be provided in these spaces.

Longevity of the provider relationship was also important. If seeing a new or temporary provider, respondents deprioritized coming out; if seeing an ongoing provider, being out was prioritized. This decision was complicated for participants with disabilities and/or chronic illnesses, who considered their continued access to quality health care before discussing trans issues. Participant 10 explained, “I want to have important conversations about hormones or getting top surgery, but I don’t want to jeopardize getting good diabetic treatment.” Participants described a double-bind: they feared being forthcoming about their gender due to risks of jeopardizing their care. As a result, potentially affirming providers were sometimes unaware of the patient’s gender or pronouns and not given the chance to use correct language. Because disclosure was risky, participants even hesitated with trusted providers: “With my primary care guy, I haven’t had this conversation. I’m 90% sure it’d go fine, but I really love the care I get there. I think that’s what makes me hesitate to even mention it” (participant 19). Overall, this theme highlights the ongoing vigilance that trans patients engage in to assess safety cues.

### Receiving Affirming vs Medically Competent Care

Gender-affirming, transition-related (whether social, legal, and/or physical), and medically competent care represent important, related aspects of health care encounters. Although the first 2 themes explored how participants assessed whether to disclose gender-related information, this theme reflects the impacts of sharing such information. Respondents spoke of having to choose between affirming and medically competent care, or even “opting out of harm” by avoiding health care altogether.

Participants described affirming care as providers prioritizing patient autonomy, believing patients’ reports, and using names and pronouns appropriately. Participant 10 recommended, “Don’t ask me for information that you’re not ready to use.” Participants reported sometimes feeling treated as inanimate objects, inconveniences, learning experiences, or medical curiosities. Participant 16 shared that their providers got “morbidly curious about how certain [gender-affirming] surgeries work[ed] or what certain medical processes look[ed] like,” while participant 8 reported feeling like “an inanimate object that they’re going to fix.” This was even more pronounced for participants from racial or ethnic minority groups: “I don’t want to be the most ‘exotic’ thing in the room, you know?” (participant 26). These interactions resulted in patients feeling unsafe, uncared for, and misunderstood.

Participants reported experiences of medical incompetence from providers who lacked experience with clients who are trans, disabled, larger-bodied, and/or a member of a racial or ethnic minority group. Participants reported having to prioritize different parts of their identities in medical spaces because they suspected their providers could not attend to all aspects of their personhood. For example, sometimes their other identities (eg, race and ethnicity, body size, disability) were more important than gender when addressing critical health care needs: “I’m disabled, and I often need to decide… is it important that I point out that I’m being misgendered? Or is it important that I receive the medical care that I need? And I feel like I’m always balancing that” (participant 10).

Given these competing demands, participants often prioritized physical health care over psychological safety. Because many providers could only provide 1 type of care, some respondents reported splitting care among multiple providers: “Doctors seem to either care about me as a trans person, or have a [medical] specialty but then not actually be trans competent. And so, I hide the fact that I’m trans and get certain care from some places and then get my trans health care from another” (participant 20).

Regarding emergency care, respondents prioritized medically competent care while voicing the desire for this care to also be gender affirming. Participant 26 explained, “In trauma, …it should just all be about making sure your patient does not die.” Others shared that being misgendered in those moments made an already difficult situation even more challenging. After experiencing a major injury, participant 19 described, “life ha[d] just turned completely upside down and I was in a lot of pain. It was almost impossible to self-advocate… If they had used my pronouns, that would have just brought a little bit more ease… [and] not made it even more traumatic.” Participant 10 reflected, “When you’re hospitalized, if they come in and use your deadname and the wrong pronouns… it puts you in a worse mental space when you’re already in a shitty situation.”

Participants also shared insights for improving health care spaces, which included increasing communication with the patient and improving provider education. Participant 16 explained how it “instantly gave me a good feeling” when their provider explained various aspects of laboratory tests, something no other provider had done. Although participants emphasized the need for better provider education, they also acknowledged that providers having “perfect” phrasing was not of utmost importance. Rather, they were more concerned with providers’ goodwill and capacity to learn. “If they’re well informed and they really see us for who we are, we’ll know… and if they don’t …we’ll know. [We know] who’s trying to do good by us, and maybe who’s learning, but still figuring it out” (participant 11).

### “How Are You Going to Fit [Me] Into Your System?”

Participants expressed that their needs as patients felt at odds with the current health care system, whether that meant they did not fit neatly into a prescribed identity label or the health care system prioritized different interventions. Participants highlighted how small shifts (such as using chosen names in medical records) could facilitate better, more seamless patient care. Participants wanted knowledge of and control over how this information was shared, particularly in electronic health records. Some participants preferred electronic health records to avoid repeatedly coming out to providers, while others did not want this information stored in such a way.

At the core of these critiques, participants highlighted how complex issues of gender are: “These 2 boxes (man or woman) have just come to represent so much more information than they really do [eg, genitals, hormone levels]” (participant 20). Participant 4 added cultural nuance to this: “The country that I’m from [has] the oldest recorded trans community in history… they’re… like blessed beings, you know? There’s a culture behind it. It’s hard to explain, but how are you going to fit that into your system?” Given such complexities, participants questioned how the current health care system could ever provide truly gender-affirming, medically competent care.

Participants criticized half-hearted attempts at inclusivity: “If you’re going to take these steps, take them all the way.” They cited examples of providers using “performative” allyship, in which providers used “correct words” but still adhered to gendered stereotypes or provided transphobic care. For participant 10, the performative allyship they have witnessed, which they called “cop-outs to make cis people feel good” and “sloppy attempts at trying to be trans inclusive” was often dated (eg, “preferred pronouns,” “transgendered”) or insincere, excluded intersex individuals, celebrated “box-checking exercises” and was emotionally upsetting. Ultimately, while acknowledging the role and impact of individual provider behaviors and steps that trans patients can take to mitigate harm, participants recognized the need for systemic changes. This recognition is reflected by participant 9, who said: “I think we get really caught up in, like, ‘Are we asking the right questions? Are we getting it right? Can you please tell us that we’re doing the right thing?’ more so than some of the deeper, real shit that people need to… and that’s not necessarily helpful. We need a larger trans health reckoning… because this is the easy stuff.” Although discussing gender-related information was often a site of uncertainty, the interviewees demonstrated deep understanding of the complexity of these interactions and the resulting impacts on their health care.

### Conceptual Model of Competent and Affirming Health Care for Trans Patients

Informed by these results, the Trans Care Bicycle (eFigure in Supplement 1) provides 1 conceptual representation of necessary factors of competent and affirming health care for trans patients. The systems level provides structure (eg, policies, systems of care, protocols, best practices, culture, political climate) that supports or hinders the presence of medically competent and gender-affirming care. It is acknowledged that people, whether individuals or groups, steer systems-level components. For trans patients to have equitable health care, the care they are provided must be both medically competent (whether pertaining to transition- or gender-related needs or not) and affirming of their gendered and intersectional experiences. Discussing their gender with health care providers is just 1 component of trans patients’ overall health care experience.

## Discussion

Our study both underscores and extends current scholarship. Study findings highlight the complex lived experiences of trans individuals as they navigate sharing gender-related information in health care spaces. Although data collection procedures and word choice were important, the specific phrasing of questions was not the most meaningful component. Rather, how gender is implicated in relevance to their health care, one’s overall safety or risk of discrimination, and authentic support of trans individuals took priority. These findings illustrate the breadth of impacts that provider behaviors have on patients’ health care experiences, not only psychologically but also as it pertained to whether participants felt able to discuss the details of their gender at all.

Although some participants avoid health care due to discriminatory treatment, others carefully choose health care providers. Although many are often well versed in trans-specific health issues, trans patients still appreciated provider health care communication. Participants preferred providers who were LGBTQ, echoing research regarding the positive effect of connecting with community.^27,28^ Participants recognized that patients rarely get to choose specialists or emergency or trauma providers, which increased stress and vulnerability during these encounters. As reflected in other studies, disabled participants mentioned that they were rarely able to avoid care and, along with participants who are larger-bodied, as well as those who belong to racial or ethnic minority groups, that they often had to prioritize safety of one identity over another.^29,30,31,32^ Findings also underscore the challenges trans patients face in emergency care, where provider-patient relationships are inherently new and the consequences of discrimination are potentially life threatening.

Participant comments regarding medical records are underscored in current recommendations.^3,33,34^ Overall, participants mentioned that intake forms set the stage for provider interactions. Dovetailing with current scholarship regarding gender-affirming care,^35^ participants indicated that “flagging” (ie, microbehaviors reflecting trans competence) increased provider trust. Participants also appreciated providers initiating these gender-related conversations (eg, by introducing themselves with their pronouns, asking respectful follow-up questions, and implementing inclusive forms).

Findings suggest that providers be responsive to patient preferences when discussing gender-related information. Providers should explain how requested information is relevant to the patient’s care and how the information will be protected. Provider education should focus not only on general trans competency but also on the importance of mirroring patient language, giving patients autonomy, and how to nimbly shift language or behavior to fit patient needs. Although education on trans issues should be mandated for all providers,^7,8,36^ training itself is not the sole solution to complex social problems. Training must be paired with systems-level changes and accountability when clinicians harm trans patients. Future scholarship should continue to leverage the expertise of trans individuals regarding their own care and reimagining of a health care system that is both competent and affirming.^37^

### Strengths and Limitations

This study has some strengths. A unique strength of this study was the diversity (ie, age, race and ethnicity, gender, ability status) of the majority trans research team, who all worked in emergency departments or with community-level programs.

This study also has some limitations. Although participants represented multiple US regions, the findings are historically and socially situated. Finally, the sample was largely White; therefore, the experiences of trans individuals who are members of racial and ethnic minority groups are less represented.

## Conclusions

In this qualitative study of trans patient preferences, when discussing gender, the 4 identified themes informed a conceptual model of competent and affirming health care for trans patients. Competent trans health care requires ongoing medical developments to meet the unique needs of trans communities, as well as updated medical education addressing patients’ physical and mental health needs. Attending to affirming language and pronouns is only part of ethical practice. Competent health practice also involves risk taking, vulnerability, humility, and patient engagement, along with organizational policies that advance health justice for trans communities.

## Notes

Supplement 1. **eFigure.** Conceptual Model: the Trans Care Bicycle

Supplement 2.
Data Sharing Statement

## References

[1] Tordoff DM, Wanta JW, Collin A, Stepney C, Inwards-Breland DJ, Ahrens K. Mental health outcomes in transgender and nonbinary youths receiving gender-affirming care. JAMA Netw Open. 2022;5(2):e220978. doi:10.1001/jamanetworkopen.2022.0978 35212746 PMC8881768

[2] van der Miesen AIR, Steensma TD, de Vries ALC, Bos H, Popma A. Psychological functioning in transgender adolescents before and after gender-affirmative care compared with cisgender general population peers. J Adolesc Health. 2020;66(6):699-704. doi:10.1016/j.jadohealth.2019.12.018 32273193

[3] Alpert AB, Mehringer JE, Orta SJ, . Experiences of transgender people reviewing their electronic health records, a qualitative study. J Gen Intern Med. 2023;38(4):970-977. doi:10.1007/s11606-022-07671-6 35641720 PMC10039220

[4] Hostetter CR, Call J, Gerke DR, Holloway BT, Walls NE, Greenfield JC. “We are doing the absolute most that we can, and no one is listening”: barriers and facilitators to health literacy within transgender and nonbinary communities. Int J Environ Res Public Health. 2022;19(3):1229. doi:10.3390/ijerph19031229 35162254 PMC8834767

[5] James SE, Herman JL, Rankin S, Keisling M, Mottet L, Anafi M. The Report of the 2015 U.S. Transgender Survey. National Center for Transgender Equality; 2016.

[6] Kachen A, Pharr JR. Health care access and utilization by transgender populations: a United States Transgender Survey Study. Transgend Health. 2020;5(3):141-148. doi:10.1089/trgh.2020.0017 33644308 PMC7906231

[7] Kattari SK, Call J, Holloway BT, Kattari L, Seelman KL. Exploring the experiences of transgender and gender diverse adults in accessing a trans knowledgeable primary care physician. Int J Environ Res Public Health. 2021;18(24):13057. doi:10.3390/ijerph182413057 34948676 PMC8701045

[8] Pulice-Farrow L, Gonzalez KA, Lindley L. “*None of my providers have the slightest clue what to do with me*”*:* transmasculine individuals’ experiences with gynecological healthcare providers. Int J Transgend Health. 2020;22(4):381-393. doi:10.1080/26895269.2020.1861574 37808533 PMC10553369

[9] Wall CSJ, Patev AJ, Benotsch EG. Trans broken arm syndrome: a mixed-methods exploration of gender-related medical misattribution and invasive questioning. Soc Sci Med. 2023;320(320):115748-115748. doi:10.1016/j.socscimed.2023.115748 36736052

[10] Kattari SK, Bakko M, Hecht HK, Kattari L. Correlations between healthcare provider interactions and mental health among transgender and nonbinary adults. SSM Popul Health. 2019;10:100525. doi:10.1016/j.ssmph.2019.100525 31872041 PMC6909214

[11] Romanelli M, Lu W, Lindsey MA. Examining mechanisms and moderators of the relationship between discriminatory health care encounters and attempted suicide among US transgender help-seekers. Adm Policy Ment Health. 2018;45(6):831-849. doi:10.1007/s10488-018-0868-8 29574543

[12] Ruppert R, Kattari SK, Sussman S. Review: prevalence of addictions among transgender and gender diverse subgroups. Int J Environ Res Public Health. 2021;18(16):8843. doi:10.3390/ijerph18168843 34444595 PMC8393320

[13] Scheim AI, Perez-Brumer AG, Bauer GR. Gender-concordant identity documents and mental health among transgender adults in the USA: a cross-sectional study. Lancet Public Health. 2020;5(4):e196-e203. doi:10.1016/S2468-2667(20)30032-3 32192577 PMC9912749

[14] McNeil J, Bailey L, Ellis S, Morton J, Regan M. Trans Mental Health Study 2012. Equality Network; 2012. Accessed July 19, 2022. https://www.scottishtrans.org/wp-content/uploads/2013/03/trans_mh_study.pdf

[15] Jackson J, Stewart AM, Fleegler EW. Down but not defeated: clinicians can harness the power of policy for LGBTQ+ rights. Prev Med. 2023;167:107423. doi:10.1016/j.ypmed.2023.107423 36641128

[16] Grant JM, Mottet LA, Tanis J, Herman JL, Harrison J, Keisling M. Injustice at Every Turn: A Report of the National Transgender Discrimination Survey. February 3, 2011. National LGBTQ Task Force. Accessed July 19, 2022. https://www.thetaskforce.org/resources/injustice-every-turn-report-national-transgender-discrimination-survey/

[17] Progovac AM, Cook BL, Mullin BO, . Identifying gender minority patients’ health and health care needs in administrative claims data. Health Aff (Millwood). 2018;37(3):413-420. doi:10.1377/hlthaff.2017.1295 29505378 PMC5942884

[18] Janesick L. “Stretching” Exercises for Qualitative Researchers. 3rd ed. SAGE; 2011. doi:10.1177/136078041101600402

[19] Creswell JW. Qualitative Inquiry and Research Design: Choosing Among Five Approaches. 3rd ed. Sage Publications; 2013.

[20] Heidegger M. Being and Time: A Translation of Sein und Zeit. Stambaugh J, trans. State University of New York Press; 1996.

[21] Zebrack BJ. Cancer survivor identity and quality of life. Cancer Pract. 2000;8(5):238-242. doi:10.1046/j.1523-5394.2000.85004.x 11898236

[22] Smith JA, Flowers P, Larkin M. Interpretative Phenomenological Analysis. Sage Publications; 2009.

[23] Harris PA, Taylor R, Minor BL, ; REDCap Consortium. The REDCap Consortium: building an international community of software platform partners. J Biomed Inform. 2019;95:103208. doi:10.1016/j.jbi.2019.103208 31078660 PMC7254481

[24] Harris PA, Taylor R, Thielke R, Payne J, Gonzalez N, Conde JG. Research electronic data capture (REDCap)—a metadata-driven methodology and workflow process for providing translational research informatics support. J Biomed Inform. 2009;42(2):377-381. doi:10.1016/j.jbi.2008.08.010 18929686 PMC2700030

[25] Dedoose. Dedoose, version 9.0.17: web application for managing, analyzing, and presenting qualitative and mixed method research data. SocioCultural Research Consultants, LLC; 2021. Accessed January 30, 2024. https://www.dedoose.com/

[26] Braun V, Clarke V. Using thematic analysis in psychology. Qual Res Psychol. 2006;3(2):77-101. doi:10.1191/1478088706qp063oa

[27] Bowling J, Barker J, Gunn LH, Lace T. “It just feels right”: perceptions of the effects of community connectedness among trans individuals. PLoS One. 2020;15(10):e0240295. doi:10.1371/journal.pone.0240295 33017435 PMC7535036

[28] Harner V. Trans intracommunity support & knowledge sharing in the United States & Canada: a scoping literature review. Health Soc Care Community. 2021;29(6):1715-1728. doi:10.1111/hsc.13276 33438797

[29] Burke TB. Choosing accommodations: signed language interpreting and the absence of choice. Kennedy Inst Ethics J. 2017;27(2):267-299. doi:10.1353/ken.2017.0018 28736422

[30] FitzGerald C, Hurst S. Implicit bias in healthcare professionals: a systematic review. BMC Med Ethics. 2017;18(1):19. doi:10.1186/s12910-017-0179-8 28249596 PMC5333436

[31] Glenn-Applegate K, Pentimonti J, Justice LM. Parents’ selection factors when choosing preschool programs for their children with disabilities. Child Youth Care Forum. 2011;40(3):211-231. doi:10.1007/s10566-010-9134-2

[32] Turcotte PL, Larivière N, Desrosiers J, . Participation needs of older adults having disabilities and receiving home care: met needs mainly concern daily activities, while unmet needs mostly involve social activities. BMC Geriatr. 2015;15(1):95. doi:10.1186/s12877-015-0077-1 26231354 PMC4522124

[33] Deutsch MB, Buchholz D. Electronic health records and transgender patients—practical recommendations for the collection of gender identity data. J Gen Intern Med. 2015;30(6):843-847. doi:10.1007/s11606-014-3148-7 25560316 PMC4441683

[34] Kronk CA, Everhart AR, Ashley F, . Transgender data collection in the electronic health record: current concepts and issues. J Am Med Inform Assoc. 2022;29(2):271-284. doi:10.1093/jamia/ocab136 34486655 PMC8757312

[35] Goldman MP, Campbell KL, Sousa D, Doolittle BR. A new skill is needed in the emergency department: introducing ourselves properly. Acad Emerg Med. 2022;29(1):131-132. doi:10.1111/acem.14414 34751968

[36] Simons L, Voss R. Advocating for transgender and gender expansive youth in the emergency setting. Clin Pediatr Emerg Med. 2020;21(2):100780. doi:10.1016/j.cpem.2020.100780

[37] Weingartner LA, Combs RM, Bohnert CA, Decker HR, Noonan EJ. Epistemic peerhood as a model to improve gender-affirming care in medical education. Teach Learn Med. Published online October 31, 2022. doi:10.1080/10401334.2022.2137169 36314249

