# Supplementary material for: Transgender Patient Preferences When Discussing Gender in Health Care Settings
Source: JAMA Netw Open. 2024 Feb 19;7(2):e2356604. doi: 10.1001/jamanetworkopen.2023.56604 (PMC10877454; doi:10.1001/jamanetworkopen.2023.56604)
Supplement: Supplement 1. — eFigure. Conceptual Model: the Trans Care Bicycle [file jamanetwopen-e2356604-s001.pdf]

Supplementary Online Content

Harner V, Moore M, Casillas B, Chrivoli J, Lopez Olivares A, Harrop E. Transgender patient preferences when discussing gender in health care settings. *JAMA Netw Open.* 2024;7(2):e2356604. doi:10.1001/jamanetworkopen.2023.56604

eFigure. Conceptual Model: the Trans Care Bicycle

This supplementary material has been provided by the authors to give readers additional information about their work.

© 2024 Harner V et al. *JAMA Network Open.*

eFigure. Conceptual Model: the Trans Care Bicycle

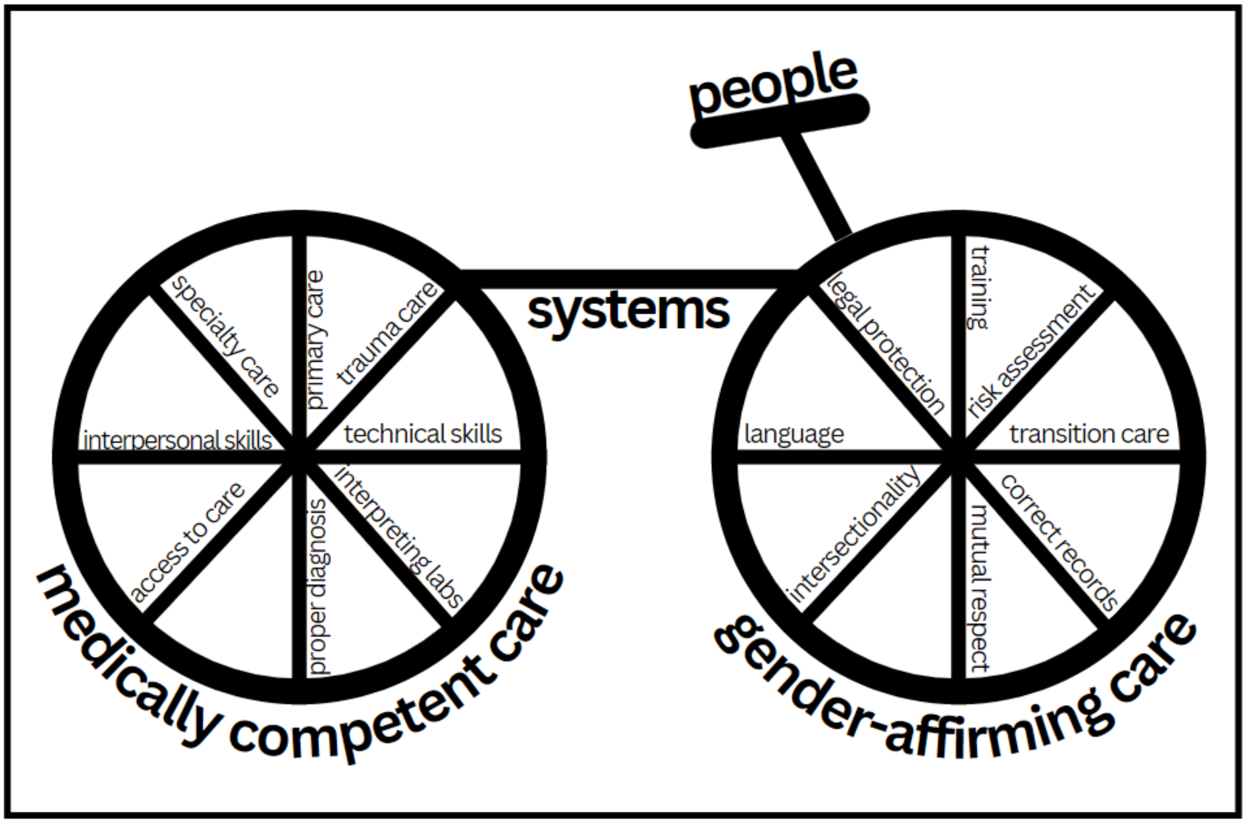

© 2024 Harner V et al. *JAMA Network Open.*
