# Supplementary material for: Transgender Patient Preferences When Discussing Gender in Health Care Settings
Source: JAMA Netw Open. 2024 Feb 19;7(2):e2356604. doi: 10.1001/jamanetworkopen.2023.56604 (PMC10877454; doi:10.1001/jamanetworkopen.2023.56604)
Supplement: Supplement 2. — Data Sharing Statement [file jamanetwopen-e2356604-s002.pdf]

Data Sharing Statement

Harner. Transgender Patient Preferences When Discussing Gender in Health Care Settings. *JAMA Netw Open*. Published February 19, 2024. doi:10.1001/jamanetworkopen.2023.56604

Data
Data available: No

Additional Information
Explanation for why data not available: Due to the vulnerable nature of the interviews and lack of consent provided by interviewees to share their full transcripts, we are unable to share the full transcripts outside of the research team.
